# SNP allele calling of Illumina Infinium Omni5-4 data using the butterfly method

**DOI:** 10.1038/s41598-022-22162-8

**Published:** 2022-10-12

**Authors:** Mikkel Meyer Andersen, Steffan Noe Christiansen, Jeppe Dyrberg Andersen, Poul Svante Eriksen, Niels Morling

**Affiliations:** 1 Department of Mathematical Sciences, Aalborg University, Aalborg, Denmark; 2 Section of Forensic Genetics, Department of Forensic Medicine, Faculty of Health and Medical Sciences, University of Copenhagen, Copenhagen, Denmark

**Keywords:** Statistics, Genetics

## Abstract

We introduce a within-sample SNP calling method, called the “butterfly method”, that improves the quality of SNP calling with the Illumina Infinium Omni5-4 SNP Kit. This was done by improving how no-calls are determined from allele signal intensities. High confidence of SNP allele calling is extremely important in forensic genetics and clinical diagnostics. This paper is accompanied by two open-source R packages, omni54manifest and snpbeadchip that make SNP calling easy by helping with bookkeeping and giving easy access to meta-information about the SNPs typed with the Illumina Infinium Omni5-4 Kit (including chromosome, probe type, and SNP bases). We compared the results from our method with those obtained with the Illumina GenomeStudio software (which does not provide sample and SNP specific genotype probabilities or other quality measures), and with whole-genome sequencing (WGS). Given the signal intensities, the SNP calling quality was optimised using a threshold for the *a posteriori* probability of a SNP belonging to a SNP cluster. By lowering the *a posteriori* probability threshold for no-calls, we obtained a higher call rate than GenomeStudio. Using a higher *a posteriori* probability threshold, we achieved a higher concordance with the WGS data than GenomeStudio. Our method had SNP call and concordance rates with WGS data of approximately 99%.

## Introduction

In forensic genetics and clinical diagnostics, high certainty of SNP allele calling is essential. Particularly in criminal cases with small amounts of DNA from crime scenes, the interpretation of DNA results is of utmost importance. The presently used and published SNP allele calling methods are reasonably reliable for optimal amounts of DNA. However, the existing SNP calling methods can be improved and adapted to handle results from suboptimal DNA amounts.

We introduce a new method for SNP calling, exemplified with data from the Illumina Infinium Omni5-4 Kit^1^. The Omni5-4 is based on the Illumina BeadChip technology that has two types of probes, type I and type II. The nucleotides are detected by two colour channels: red (detects A/T nucleotides) and green (detects C/G nucleotides). Probe type I has two bead addresses such that one nucleotide is interrogated by the beads with one address and the other nucleotide by the beads with the other address. The light emitted by the fluorophores attached to the probes are measured by the same colour channel on both bead addresses by the design of the probe sequences, i.e., either red or green. The other colour channel is not used for this probe type but can potentially be used for estimating, e.g., the background noise. The probe type II has only one bead address such that the signal from one nucleotide is measured by the red colour channel and the other nucleotide is measured by the green colour channel. The output from the array is the mean signal intensity from each colour channel together with the standard deviation and number of beads investigated.

The Illumina method calls one or two alleles that—to some confusion—are called A/B alleles^2,3^. Simplified, the A/B system tells whether the SNP position has homozygous reference alleles, homozygous alternative alleles, or heterozygous alleles. The A/B alleles must be converted to the standard DNA bases A, T, G, and C using a manifest file^4^ created by Illumina.

Bookkeeping of the selection of the right colour channel for the right probe and conversion of the A/B allele system to the standard DNA bases is a tedious task with many different rules that must be applied. We have developed open-source software that can help do these tasks. The software is published as an R^5^ package called snpbeadchip^6^ for selecting the correct colour channels and converting the A/B alleles to plus/minus alleles and an accompanying R package called omni54manifest^7^ that provides easy access to the information about the probes such as the manifest^4^ and mapping information^8^. snpbeadchip uses illuminaio^9^ to read idat files.

Once the signal intensities of the reference and alternative alleles are obtained, the SNP can be called. We propose a method referred to as the “butterfly method” (cf. Fig. 2).

We tested the butterfly method against high-quality PCR-free (shotgun) whole-genome sequencing (WGS) data, which are considered the gold-standard for “concordance”. Furthermore, we compared the butterfly method with SNP calls from the Illumina GenomeStudio software^10^.

The aims of this paper and the purpose of the method are to provide an open description of a SNP calling method with transparent handling of no-calls that others can use, replicate, and improve. The description of the method used by GenomeStudio called GenTrain 3.0^11^ is not publicly available^12^. It uses the data of the other samples to analyse the sample in question, which can be problematic because the SNP calling of a sample should for this kind of analysis, in principle, not be influenced by other samples. One such problematic situation may arise if the samples analysed together are of varying quality, e.g., the combination of samples with high quality and partly degraded DNA, as may be the case in a forensic setting. Also, the GenTrain does not provide *a posteriori* genotype probabilities for each SNP of a sample. Hence, there is no obvious way to adjust the no-call algorithm. The GenTrain Score is given for each SNP and is a combined score for all samples.

## Materials and methods

All statistical analyses were made using R^5^ version 4.1.2 and tidyverse^13^.

### Ethics

The study was approved by the Committees on Health Research Ethics in the Capital Region of Denmark (H-2-2012-017). The biobank where the samples are held is registered at the University of Copenhagen’s joint records of processing of personal data in research projects and biobanks (514-0725/22-3000) and complies with the rules of the General Data Protection Regulation (Regulation (EU) 2016/679). According to The Danish National Committee on Health Research Ethics, informed consent is not necessary for the samples used in this study (H-2-2012-017). The study was performed in accordance with the ethical standards as laid down in the 1964 Declaration of Helsinki and its later amendments and comparable ethical standards.

### Blood samples and DNA extraction

Peripheral blood samples from three individuals were collected and stored at − 20 °C until DNA extraction. DNA extraction was carried out using the DNeasy Blood & Tissue Kit (Qiagen), following the manufacturer’s recommendations for purification of total DNA from whole blood.

### SNP typing using the Illumina Infinium Omni5-4 kit

All samples were analysed using the Illumina Infinium Omni5-4 Kit following the manufacturer’s recommendations with varying DNA amounts. The DNA concentration was measured using the Qubit dsDNA HS Assay Kit (Thermo Fisher Scientific). Two-fold serial dilutions of DNA from the three samples were performed using nuclease-free water to obtain samples with the following DNA amounts: 400 ng, 200 ng, 100 ng, 50 ng, and 25 ng. Briefly, the DNA was hybridised to the probes attached to the BeadChips. Hereafter, the attached probes were subject to single-base extension and stained. The BeadChips were scanned using the iScan^™^ system (Illumina) following the manufacturer’s recommendations.

### PCR-free (Shotgun) whole genome sequencing

Samples were sequenced with the NextSeq500 platform (Illumina, USA) using paired-end sequencing (2 $\times$ 150 bases). PCR-free WGS and variant detection were carried out as described in^14^. AdapterRemoval version 2.1.3^15^ identified and removed adapter sequences from the reads using the collapsed option. Consecutive stretches of low-quality bases ($Q<30$) were removed from the 5’ and 3’ termini, and reads shorter than 30 bases were discarded. The Phred+33 quality scores encoding was used. For alignment, we used BWA-MEM version 0.7.10-r789^16^ and accepted only properly aligned reads (samtools flag -f 0x2). GATK version 4.0.0.0 with HaplotypeCaller^17^ with standard settings was used for variant calling. The reads were approximately normally distributed with a mean coverage of 37, and we only used biallelic SNPs with at least 25 reads.

### The butterfly method

“The butterfly method” is based on a finite mixture of bivariate normal distributions^18^ with three mixture components: one for each SNP/genotype, i.e., AA, AB, or BB in the A/B allele system^2^.

Let *A* and *B* be the mean signal intensities for alleles A and B in the A/B allele system, respectively. We $\log$ transformed using the natural logarithm of the mean signal intensities with one added to avoid numerical problems as the mean signal intensity can be 0, and $\ln (0) = - \infty$. Thus, we used $A' = \ln (A+1)$ instead of *A* in the models.

Using $A' = \ln (A+1)$ and $B' = \ln (B+1)$, the model specifies a joint probability density function by

1 $$\begin{aligned} f(A', B') = \sum _{i=1}^3 \tau _i \phi _i(A', B') \end{aligned}$$

where $i \in \{1, 2, 3\}$ indicates the SNP group (e.g., $i = 1$ means AA, $i = 2$ means AB, and $i = 3$ means BB), $\phi _i$ is a probability density function for a bivariate normal distribution, and $\tau _i = P(i)$ is the *a priori* (without taking intensities $A'$ and $B'$ into account) probability that the SNP has type *i* (and $\sum _{i=1}^3 \tau _i = 1$).

In other words, we model the signal intensities as a three-component mixture of bivariate normal distributions. The signal intensities $A'$ and $B'$ can either come from SNP group 1 (AA), 2 (AB) or 3 (BB). The likelihoods of observing $A'$ and $B'$ in each SNP group are weighted by $\tau _i$.

The unknown parameters in the model include, e.g., the *a priori* probabilities, $\tau _i$, the mean values, and the covariance matrices for the bivariate normal distributions (not shown). The parameters were estimated using the R package mclust^18^. We chose to model the mixture components as bivariate normal with any shape and orientation; in the mclust terminology, this is called a VVV model.

When calling SNPs, we wanted to calculate the *a posteriori* probability of SNPs belonging to SNP group *k*, given the signal intensities $A'$ and $B'$, which is given by

2 $$\begin{aligned} P(k \mid A', B') = \frac{P(k , A', B')}{P(A', B')} = \frac{P(A', B' \mid k) P(k)}{\sum _{i=1}^3 P(A', B' \mid i) P(i)} = \frac{\tau _k \phi _k(A', B')}{ \sum _{i=1}^3 \tau _i \phi _i(A', B') } . \end{aligned}$$

The *a posteriori* probability, $P(k \mid A', B')$, is the *a priori* probability of being in group *k*, $\tau _k$, regardless of the allele intensities $A'$ and $B'$, multiplied by the likelihood of the allele intensities in SNP group *k*, $\phi _k(A', B')$, and normalised (denominator) so that the sum of the *a posteriori* probabilities of the three SNP groups is 1.

We present three variants of the butterfly method. Different data sets were used to train (estimate the parameters of) the three-component mixture model: 1) each sample was its own reference using all SNPs simultaneously, 2) like 1, except using separate models for the two probe types (I/II), and 3) an ensemble model using all samples to estimate a single model.

#### SNP calling

We used the recommended settings in GenomeStudio^10^ and the Genotyping Project Wizard to import sample data into Genome Studio based on sample intensities (not based on existing cluster files) and the “Cluster All SNPs” function under the “Analysis” tab in GenomeStudio to generate SNP clusters.

For the butterfly method, we called the SNP genotype with the maximal *a posteriori* threshold, except for situations with no-calls (NC). We chose always to make a NC if the mean signal intensities for both alleles A and B were 0.

We investigated two ways of making a NC. Firstly, if the maximal *a posteriori* probability was below a certain threshold, we made a NC. This was done for a range of thresholds (from 0.5 to 0.999). Secondly, we chose to consider the number of beads with which the SNPs had been investigated, and on which the mean signal intensities were based. If the number of beads was below five, we made a NC. We also used a threshold of zero beads.

For probe type II, the same beads capture both alleles, so there is only one number of beads for each investigated position. For probe type I, different beads capture each allele, so there is a certain number of beads for allele A and another one for allele B. For probe type I, both numbers of beads must be above the threshold.

Imposing such NC thresholds results in calling fewer alleles but with higher confidence in the alleles called.

#### Other methods

The GenoSNP method introduced in^19^ is a within-sample method that uses a four-component mixture of *t*-distributions (like the normal distribution, but with heavier tails), where the fourth cluster is a “null class” for capturing outliers. The calls are made by identifying the cluster with the maximal *a posteriori* probability. Hence, the no-calls are selected when the null class has the highest *a posteriori* probability. The classification of the outliers in a single “null class” is problematic as the outliers do not behave in the same way. Outliers are not expected to be distributed according to a *t*-distribution and grouped in the same cluster in the $(A', B')$ space.

The M3 method introduced in^20^ is similar to that in^19^, except that a four-component mixture of normal distributions is used and the focus is on calling rare variants. In^20^, the *a posteriori* probability is mentioned, but only in connection with calculating the average *a posteriori* probability for each SNP.

To summarise, our paper contributes the following novel work: a) analysis of data obtained with the Illumina Infinium Omni5-4 Kit by comparing SNP calls made by GenomeStudio and GenTrain 3.0^10,11^; b) demonstrating how the *a posteriori* probabilities and the numbers of beads can be used to categorise NC (instead of including an unrealistic null-cluster) and analyse how they impact the concordance with WGS calls; c) showing how a non-sample specific, a general model, and a sample and probe type specific model perform. Our method is available as the R software packages snpbeadchip^6^, omni54manifest^7^, and the existing R software package mclust^18^ for estimating the mixture model with the function mclust(..., G = 3, modelNames = "VVV") and calculating *a posteriori* probabilities with the function predict().

We chose not to include all the above methods because the main focus of this paper was to explore the possibility of adjusting the NC rate and offering open-source software for this purpose.

## Results

The Omni5-4 manifest^7^ has 4,327,108 SNPs. We removed 271,680 SNPs (details in Table 1) and ended up with 4,055,428 autosomal SNPs (93.7% of the original). Of the 4,055,428 SNPs included, 135,419 SNPs were typed with type I probes (ambiguous), and 3,920,009 were typed with type II probes (unambiguous).

The mclust^18^ function with the *VVV* model on the object x (i.e., mclust(x, G = 3, modelNames = "VVV")) took about 3 minutes to run per sample (4,055,428 SNPs) on a AMD EPYC 7351 16-Core Processor. The x object contained the $\log$ transformed signal intensities in its two columns for all 4,055,428 SNPs, and it occupied 62 MB memory in R.

**Table 1:** Filtering of probes.

Steps	Description	SNPs removed	SNPs left
0	Manifest file		4,327,108
1	Type II and ambiguous (rs28362918, rs28897688)	2	4,327,106
2	Chromosome 0 (probe problems)	9724	4,317,382
3	Chromosome X/Y/mitochondria	121,387	4,195,995
4	Non-empty mapping comment (probe problems)	636	4,195,359
5	INDELs (insertion-deletions)	4412	4,190,947
6	Multiple probes binding to same rsID	129,739	4,061,208
7	Multiple rsIDs for a name	5780	4,055,428 (93.7%)

The filtering was performed sequentially in the order shown. The numbers are conditioned on the filtering order. Steps 2 and 4 were performed to remove probes with registered problems of various kinds.

**Figure 1:**
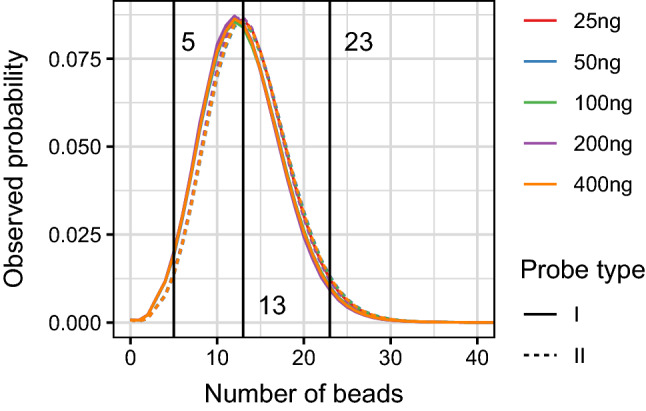
The densities of the numbers of beads for both probe types I and II from three individuals. There is one curve for each probe type and dilution, 10 in total. The densities are almost similar. The interval [5, 23] is the middle 95% of the probability mass for both probe types and all dilutions. Hence, 95% of the probes were expected to have between 5 and 23 beads. The median number of probes for both probe types and all DNA concentrations was 13.

The densities of the numbers of beads for both probe types for the three individuals are given in Fig. 1.

**Figure 2:**
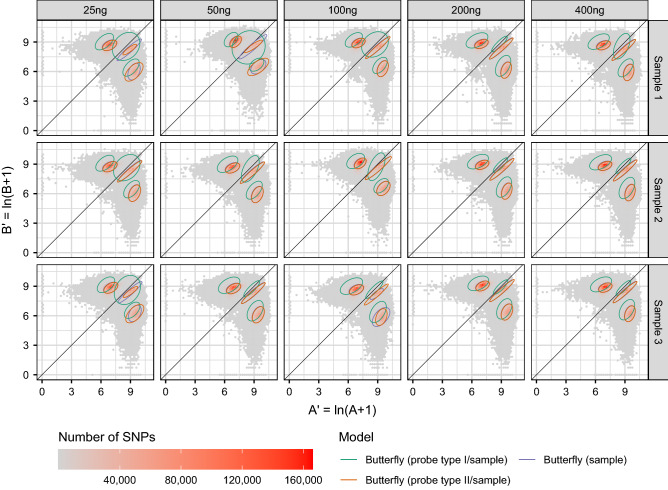
The mean signal intensities for alleles A and B. The three SNP groups AA, AB, and BB (in A/B nomenclature) form butterfly patterns. The 75% confidence ellipses of the three models were plotted on top of the transformed signal intensities. Note that the two models “Butterfly (probe type I/sample)” and “Butterfly (probe type II/sample)” together give “Butterfly (probe type/sample)”, which is used as a separate method below. The SNP groups (genotypes) are well-separated in most cases.

The mean signal intensities with an illustration of the models are shown in Fig. 2.

We only used biallic SNPs and alleles that were called with a read depth of at least 25 with WGS. This resulted in 3,139,554 SNPs called (77.42% of 4,055,428) for Sample 1; 3,875,844 SNPs called (95.57% of 4,055,428) for Sample 2; and 3,972,024 SNPs called (97.94% of 4,055,428) for Sample 3. We considered these SNP calls reliable.

Focusing on only SNPs reliably called with WGS, we calculated the concordance rates as described below.

**Figure 3:**
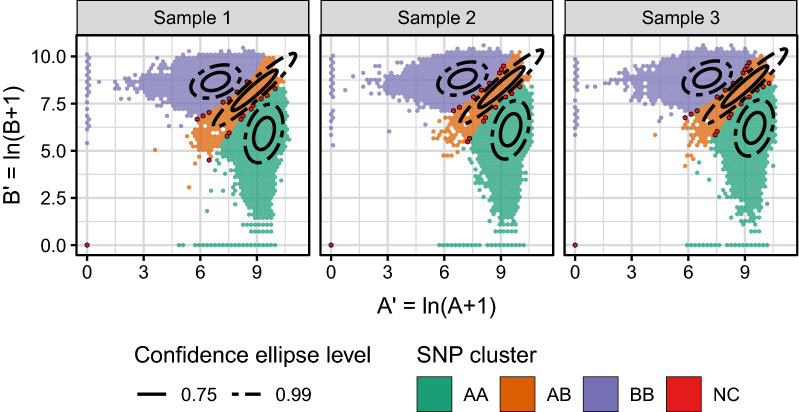
The SNP calling for the “Butterfly (sample)” method illustrated for samples with 400 ng DNA. An *a posteriori* probability threshold of 0.8 was applied for NC. Note, that the NCs are in the regions between two SNP groups and at (0, 0), and the NC regions are small compared to those of the SNP groups.

The SNP calling is illustrated in Fig. 3 for 400 ng DNA with the “Butterfly (sample)” method and an *a posteriori* probability threshold for NC of 0.8.

**Figure 4:**
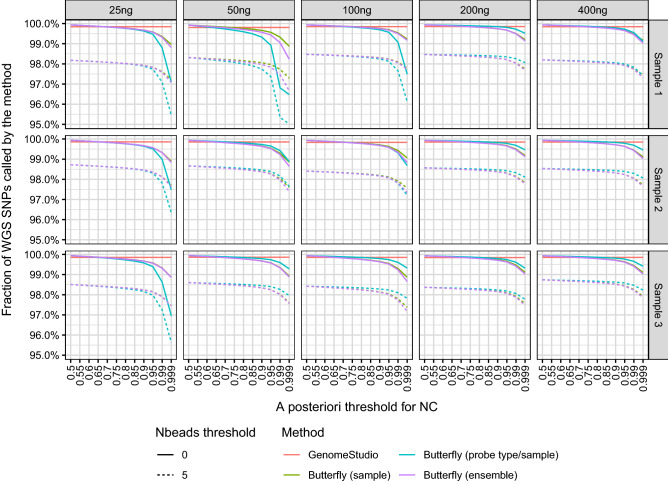
Fraction of SNP calls with the three variants of the butterfly method and GenomeStudio. The butterfly method gave a no-call (NC) if either the maximal *a posteriori* threshold of belonging to a SNP cluster was too low or if the mean signal intensity of either allele A or B (A/B nomenclature) was based on too few beads (both thresholds shown). The call-rate was always above 95% and for *a posteriori* thresholds below 99%, the call-rate were very often above 98%.

Of the SNPs called by WGS, the number of SNPs called by the other methods are given in Fig. 4.

**Figure 5:**
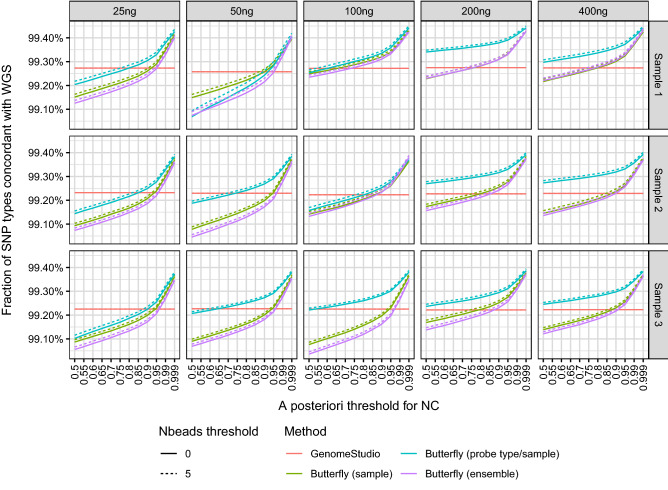
Concordance between SNP calls obtained with WGS, GenomeStudio, and the butterfly method. Only SNPs with SNP calls by all methods were included. The concordances were above 99% for all methods.

For the SNPs with WGS-based SNP calls (i.e., not NC) and SNP calls with other methods (i.e., not NC), the concordances between the WGS call and the methods were calculated (Fig. 5). The figure shows how reliable a method’s calls are when the NCs are excluded.

If one accepts fewer calls by increasing the *a posteriori* probability threshold and the number of beads threshold, the calls will be more reliable (Figs. 4 and 5).

**Figure 6:**
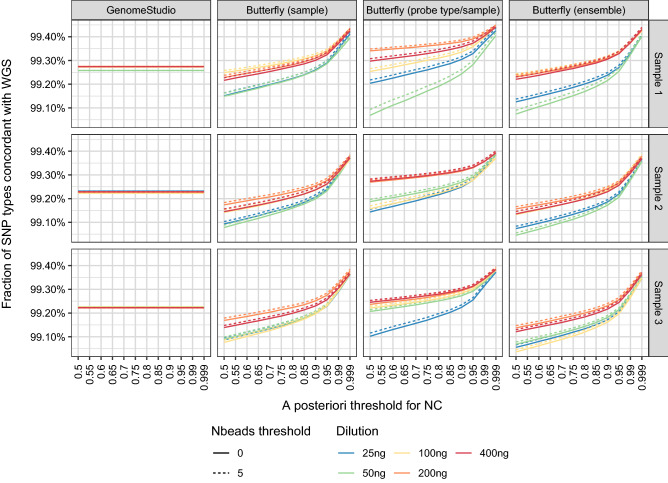
Concordance of SNP calls obtained with WGS, GenomeStudio, and the butterfly method for various DNA dilutions. Only SNPs with SNP calls by all methods were included. The figure is based on the same data as Fig. 5 but presented such that it is easier to compare the various DNA dilutions. The concordances were above 99% for all methods.

The importance of the DNA amount for choosing an *a posteriori* probability threshold can be seen in Fig. 6. For a fixed concordance, the *a posteriori* threshold must be increased the smaller the DNA amount. The lines did not follow the DNA amount ordering, possibly due to saturation and/or quantification errors, but the 25 ng and 50 ng DNA lines were often below those of 200 ng and 400 ng DNA.

**Figure 7:**
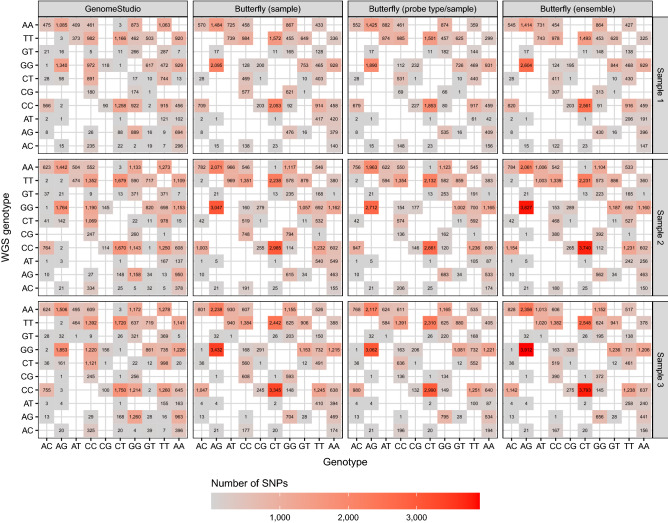
Discordant calls (excluding no-calls) for the samples with 400 ng DNA. The butterfly methods had an *a posteriori* probability threshold of 0.8 and a number of beads threshold of 0. See Fig. 8 for results for all genotypes. There is no obvious structure in these discordant calls, except possibly a small indications that all methods seem to call too few homozygous genotypes (Fig. 8).

**Figure 8:**
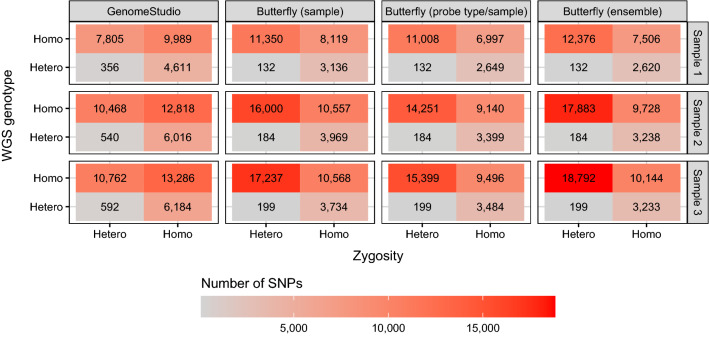
Discordant calls (excluding no-calls) for the samples with 400 ng DNA in Fig. 7 summarised to homozygous and heterozygous calls. The butterfly methods had an *a posteriori* probability threshold of 0.8 and a number of beads threshold of 0. See Fig. 7 for results for all genotypes. There is no obvious structure in these discordant calls, except possibly a small indication that all methods seem to call too few homozygous genotypes.

An overview of the discordant calls (excluding no-calls for both WGS and the methods) for 400 ng DNA is shown in Fig. 7, and homo- and heterozygous allele calls are summarised in Fig. 8.

Based on Figs. 7 and 8, it seems likely that the butterfly method’s discordances are due to heterozygous calling when WGS called homozygous. Thus, calling AG instead of GG, CT instead of CC, CT instead of TT, and AG instead of AA. A similar pattern was seen with GenomeStudio but not to the same degree. GenomeStudio made more homozygous discordancies, e.g., AA instead of TT, TT instead of AA, CC instead of GG, etc.

An overview of the discordant calls (excluding no-calls for both WGS and the methods) for 400 ng DNA is shown in Fig. 7, and homo- and heterozygous allele calls are summarised in Fig. 8.

**Figure 9:**
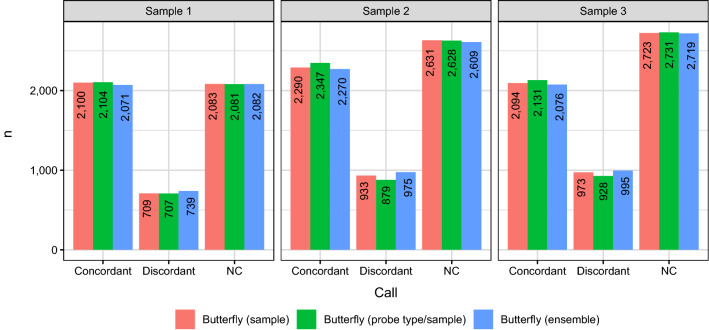
Call distribution conditional on GenomeStudio making a NC for the samples with 400 ng DNA. The butterfly methods had an *a posteriori* probability threshold of 0.8 and a number of beads threshold of 0. The butterfly method also makes approximately 50% of these NC. Given a call is made, the calls of the butterfly and WGS methods are concordant in approximately 2/3 of calls.

Figure 9 shows the no-call distribution with GenomeStudio of samples with 400 ng DNA.

## Discussion

Of the SNPs called with WGS, the butterfly method called more than 99.5% unless high thresholds for the *a posteriori* probability and number of beads were used (cf. Fig. 4). The SNP calling concordance between the butterfly method and WGS was 99.0–99.5% (Fig. 5).

We began with 4,055,428 SNPs (Table 1). Extrapolating from this (99.5%) with the uncertainty involved, we expected the butterfly method to make SNP calls of approximately 4,035,151 SNPs and no-calls for the remaining 20,277 SNPs. Of the called SNPs, 3,994,799–4,010,975 SNPs had reliable calls and 20,176–40,352 SNPs had no-calls. This gives a concordant call-rate of all SNPs of around $0.99^2$ = 98%, not taking the uncertainty into account. This emphasises that the numbers are adjustable by the two proposed thresholds (*a posteriori* probability and number of beads). The adjustment is easily done using the R packages mclust^18^ and snpbeadchip^6^.

The importance of the DNA amount and the choice of the *a posteriori* probability threshold can be seen in Fig. 5, which shows that for a fixed concordance, the *a posteriori* threshold should generally be increased for smaller DNA amounts.

We did not inspect the SNP calls from GenomeStudio and the butterfly method individually, except for excluding some SNP data at the preliminary filtering stage (Table 1). However, we believe the butterfly method’s posterior probabilities together with signal standard deviation, number of beads, etc. will be helpful in individual inspection.

Improving the SNP calling is a topic of future research. There are many ways to improve the SNP calling method proposed here. Using the WGS calls as a reference, a natural next step of modelling is discriminant analysis based on Gaussianity in a supervised learning setting. Including more explanatory variables would also enable more advanced statistical learning methods. In this study, we did not include signal variance information, which may improve SNP calling. Another option is to use probe information like base composition, colour channel, neighbour bases, etc., as explanatory variables/features. This may enable statistical learning methods like multinomial logistic regression, random forests, and deep learning.

## Conclusion

We introduced the “butterfly method” for SNP allele calling with the Illumina Infinium Omni5-4 Kit^1^ without using Illumina’s GenomeStudio software^10^. The method is a within-sample method and does not use other samples or population frequencies to call the SNP alleles. The butterfly method is based on a three-component mixture of normal distributions, in which parameters are easily estimated using the open-source statistical software R. The method is transparent, it is straight-forward to change the parameters according to the user’s needs, and easy to analyse the data within R after SNP calling. We have published two open-source R packages, omni54manifest^7^ and snpbeadchip^6^, that make SNP calling easy by helping with bookkeeping and giving easy access to meta-information about the SNPs typed with the Illumina Infinium Omni5-4 Kit (including chromosome, probe type, and SNP bases).

We tested our method on > 4 mio. SNPs and compared the results with those obtained with the GenTrain method used by Illumina GenomeStudio as well as SNPs obtained by PCR-free (shotgun) WGS. We demonstrated two variants of our method: one where we take into account potential probe type bias by estimating a separate model for each probe type (type I and type II) and another model that uses a general model such that the model’s parameter estimates do not depend on the sample that is being analysed. We focused on varying the no-call rate and showed how it changed the concordance with that of WGS. This was done by using a threshold on the *a posteriori* probability of belonging to a SNP cluster and by using the number of beads to adjust the stringency of the no-call mechanism.

With the butterfly method, we achieved a SNP call rate of around 99% and a SNP concordance with the WGS data of around 99%. By lowering the *a posteriori* probability threshold for no-calls, we obtained a higher call rate than GenomeStudio, and by increasing the *a posteriori* probability threshold, we achieved a higher concordance with the WGS data than GenomeStudio.

## Data Availability

The datasets generated during and/or analysed during the current study are available from the corresponding author on reasonable request.
